# Cardiac magnetic resonance imaging parameters show association between myocardial abnormalities and severity of chronic kidney disease

**DOI:** 10.3389/fcvm.2022.1053122

**Published:** 2022-11-17

**Authors:** Xi Jia, Xiaoyu Han, Yuqin Wang, Fangfang He, Xiaoyue Zhou, Yuting Zheng, Yingkun Guo, Rong Xu, Jia Liu, Yumin Li, Jin Gu, Yukun Cao, Chun Zhang, Heshui Shi

**Affiliations:** ^1^Department of Radiology, Union Hospital, Tongji Medical College, Huazhong University of Science and Technology, Wuhan, China; ^2^Hubei Province Key Laboratory of Molecular Imaging, Wuhan, China; ^3^Department of Nephrology, Union Hospital, Tongji Medical College, Huazhong University of Science and Technology, Wuhan, China; ^4^MR Collaborations, Siemens Healthineers Digital Technology (Shanghai) Co., Ltd., Shanghai, China; ^5^Department of Radiology, West China Second University Hospital, Sichuan University, Chengdu, China

**Keywords:** chronic kidney disease, cardiac magnetic resonance, hemodialysis, myocardial abnormalities, fibrosis, oedema

## Abstract

**Background:**

Chronic kidney disease patients have increased risk of cardiovascular abnormalities. This study investigated the relationship between cardiovascular abnormalities and the severity of chronic kidney disease using cardiac magnetic resonance imaging.

**Methods:**

We enrolled 84 participants with various stages of chronic kidney disease (group I: stages 1–3, *n* = 23; group II: stages 4–5, *n* = 20; group III: hemodialysis patients, *n* = 41) and 32 healthy subjects. The demographics and biochemical parameters of the study subjects were evaluated. All subjects underwent non-contrast cardiac magnetic resonance scans. Myocardial strain, native T1, and T2 values were calculated from the scanning results. Analysis of covariance was used to compare the imaging parameters between group I-III and the controls.

**Results:**

The left ventricular ejection fraction (49 vs. 56%, *p* = 0.021), global radial strain (29 vs. 37, *p* = 0.019) and global circumferential strain (-17.4 vs. −20.6, *p* < 0.001) were significantly worse in group III patients compared with the controls. Furthermore, the global longitudinal strain had a significant decline in group II and III patients compared with the controls (-13.7 and −12.9 vs. −16.2, *p* < 0.05). Compared with the controls, the native T1 values were significantly higher in group II and III patients (1,041 ± 7 and 1,053 ± 6 vs. 1,009 ± 6, *p* < 0.05), and T2 values were obviously higher in group I-III patients (49.9 ± 0.6 and 53.2 ± 0.7 and 50.1 ± 0.5 vs. 46.6 ± 0.5, *p* < 0.001). The advanced chronic kidney disease stage showed significant positive correlation with global radial strain (*r* = 0.436, *p* < 0.001), global circumferential strain (*r* = 0.386, *p* < 0.001), native T1 (*r* = 0.5, *p* < 0.001) and T2 (*r* = 0.467, *p* < 0.001) values. In comparison with the group II patients, hemodialysis patients showed significantly lower T2 values (53.2 ± 0.7 vs. 50.1 ± 0.5, *p* = 0.002), but no significant difference in T1 values (1,041 ± 7 vs. 1,053 ± 6).

**Conclusions:**

Our study showed that myocardial strain, native T1, and T2 values progressively got worse with advancing chronic kidney disease stage. The increased T1 values and decreased T2 values of hemodialysis patients might be due to increasing myocardial fibrosis but with reduction in oedema following effective fluid management.

**Trial registration number:**

ChiCTR2100053561 (http://www.chictr.org.cn/edit.aspx?pid=139737&htm=4).

## Introduction

Chronic kidney disease (CKD) is a major health problem worldwide with the rates of morbidity and mortality increasing by 29.3 and 41.5%, respectively, between 1990 and 2017 (1). The risk of cardiovascular disease (CVD) has been shown to be higher in CKD patients compared with the general population (2). CVD is also the leading cause of mortality in the CKD patients (3, 4). Izamura et al. (5) reported that lower estimated glomerular filtration rates (eGFRs) in CKD patients were associated with increased cardiac cell enlargement, cardiac hypertrophy, and fibrosis of the left ventricle. Therefore, CKD patients should be regarded as a high-risk group for CVD and need close medical attention at an individual level (6).

Non-contrast cardiac magnetic resonance (CMR) imaging can be used to monitor changes in myocardial mass and biventricular volumes in CKD patients undergoing long-term hemodialysis (HD) (7). CMR is also used to directly and non-invasively estimate the pathologic changes in the cardiac structure and function based on myocardial T1 and T2 mapping (8). Left ventricular (LV) strain parameters are more sensitive than left ventricular ejection fraction (LVEF) in detecting early cardiac dysfunction because they directly estimate the movement of myocardial fibers (9).

Previous CMR studies (10, 11) reported myocardial abnormalities in patients with early and advanced CKD or end-stage renal disease (ESRD) patients. These results showed that cardiac abnormalities could occur in both early-stage and advanced-stage CKD patients. Furthermore, study by Hayer et al. (12) showed that myocardial fibrosis evaluated by native T1 time was inversely associated with kidney function. However, the stage of CKD at which obvious myocardial abnormalities appear is not well-defined. Furthermore, the majority of the ESRD patients undergo HD (13, 14). However, it is not clear about the relative contributions of myocardial fibrosis to the change of native T1 times in HD patients. Therefore, in this study, we investigated the association between obvious myocardial abnormalities and CKD stages by comparing the non-contrast CMR parameters. Furthermore, we analyzed the alterations of CMR parameters in patients following HD.

## Materials and methods

### Study subjects

In this prospective longitudinal observational study, 84 participants with different stages of CKD were enrolled from the Department of Nephrology, Wuhan Union Hospital, between March 2021 to October 2021. We also enrolled 32 healthy subjects of similar age, gender, and body mass index (BMI) from the Wuhan community between March 2021 to October 2021. This study was approved by the Ethics committee of the Tongji Medical College of Huazhong University of Science and Technology. It was first registered on 24/11/2021 with the registration number ChiCTR2100053561, and was conducted in accordance with the Helsinki Declaration. The written informed consent was obtained from all the study subjects.

The inclusion criteria for participants with CKD were as follows: (1) clinically confirmed CKD at different stages; (2) age between 30 and 80 years; (3) absence of chest pain, dyspnea, and palpitations; (4) absence of history for cardiovascular diseases such as congenital heart disease, coronary artery disease, valvular heart disease, or cardiomyopathy; and (5) normal electrocardiographic manifestations. All dialysis patients had maintained hemodialysis for 4 hours, 3 times a week for at least 3 months. The inclusion criteria for the healthy controls were as follows: (1) age between 30 and 80 years; (2) absence of history for cardiovascular diseases, hypertension, hyperlipidemia, and diabetes; (3) normal physical examination; and (4) normal electrocardiographic manifestations. The exclusion criteria for the participants with CKD were as follows: (1) history of known specific cardiomyopathies, valvular heart disease or myocarditis; and (2) standard contraindications to CMR (e.g., metal implants, severe claustrophobia, and inability to hold breath).

Participants with CKD were divided into the following three groups: group I (CKD stage 1–3 patients, eGFR 30 to 120 ml/min/1.73 m^2^; *n* = 23), group II (CKD stage 4–5 patients without undergoing HD, eGFR <30 ml/min/1.73 m^2^; *n* = 20), and group III (CKD stage 4–5 patients with stable HD; *n* = 41). The estimated GFR was calculated using the four-variable Modification of Diet in Renal Disease (MDRD) formula (15) (GFR = 175 × standardized S${}_{\text{cr}}^{-1.154}$ × age^−0.203^ × 1.212 [if black] × 0.742 [if female]). Additionally, group II (CKD 4–5 non-HD patients) had very advanced CKD as evident by creatinine >500, hemoglobin 79 and calcium 1.9.

### Anthropometric and biochemical assessments

We collected data regarding sex, age, BMI, and heart rate (HR) of all the study subjects. We also extracted the values of systolic blood pressure (SBP), diastolic blood pressure (DBP), and serum biochemical parameters such as creatinine, hemoglobin, albumin, alkaline phosphatase (AKP), parathyroid hormone (PTH), urea nitrogen, uric acid, erythrocyte sedimentation rate (ESR), thyroid-stimulating hormone (TSH), free triiodothyronine (FT3), free tetraiodothyronine (FT4), triglycerides (TG), total cholesterol (TC), lactate dehydrogenase (LDH), calcium, and phosphorus for all patients from the electronic records before CMR scanning (Table 1). Study subjects were diagnosed with hypertension when the average SBP value was >140 mmHg. BMI was calculated by dividing dry weight (kg) by body height (m)^2^.

**Table 1:** Clinicopathologic characteristics of the study subjects.

**Variables**	**Healthy subjects (*n* = 32)**	**Participants with CKD (*****n*** = **84)**			***p*-values^°^**
		**Group I (CKD 1–3, *n* = 23)**	**Group II (CKD 4–5, *n* = 20)**	**Group III (HD, *n* = 41)**	
Age (years)	55 ± 11	48 ± 14^c^	49 ± 8^c^	54 ± 12^a, b^	0.336
Male (%)	15 (46.9%)	13(56.5%)	12(60%)	25(61%)	0.220
BMI (kg/m2)	23.7 ± 2.3	23.8 ± 2.7^c^	22.3 ± 2.9	22.1 ± 2.8^a^	0.064
HR (bpm)	68 ± 12	62 ± 13^b, c^	72 ± 13^a^	74 ± 10^a^	0.205
SBP (mmHg)	-	137 ± 21^c^	143 ± 19^c^	154 ± 21^a, b^	-
DBP (mmHg)	-	86 ± 14	94 ± 14^c^	81 ± 13^b^	-
Hypertension (*n*, %)	-	13(56.5%)	16(80%)	24(58.5%)	-
Diabetes (*n*, %)	-	6(26.1%)	2(10%)	9(22.0%)	-
**Serum biochemistry**					
Creatinine (μmol/L)	-	152 ± 130^b, c^	527 ± 269^a, c^	752 ± 273^a, b^	-
Hemoglobin (g/l)	-	115 ± 25^b, c^	79 ± 15^a, c^	96 ± 19^a, b^	-
Albumin (g/l)	-	31 ± 9^b, c^	35 ± 5^a^	38 ± 8^a^	-
ALK (U/L)	-	68 ± 24	62 ± 24^c^	86 ± 48^b^	-
PTH (pg/ml)	-	91 ± 55^b, c^	311 ± 258^a^	411 ± 159^a^	-
Urea nitrogen (mmol/l)	-	9 ± 5^b, c^	27 ± 11^a, c^	18 ± 7^a, b^	-
Uric acid (μmol/l)	-	398 ± 128	488 ± 166^c^	345 ± 123^b^	-
ESR (mm/h)	-	30 ± 28	35 ± 27	35 ± 36	-
FT4 (pmol/L)	-	11.1 ± 2.0^c^	12.3 ± 7.0	13.0 ± 1.4^a^	-
FT3 (pmol/L)	-	3.5 ± 0.8	3.0 ± 0.6	3.2 ± 0.5	-
TSH (μlU/ml)	-	4 ± 3	4 ± 4	5 ± 6	-
TC (mmol/L)	-	5.1 ± 1.7^b^	3.9 ± 0.8^a^	4.2 ± 1.0	-
TG (mmol/L)	-	1.6 ± 0.9	1.4 ± 0.7	1.5 ± 1.0	-
LDH (U/L)	-	226 ± 41	264 ± 97	265 ± 103	-
Calcium (mmol/L)	-	2.1 ± 0.2^c^	1.9 ± 0.3^c^	2.2 ± 0.2^a, b^	-
Phosphorus (mmol/L)	-	1.1 ± 0.2^b, c^	1.6 ± 0.4^a^	1.4 ± 0.4^a^	-

° *p*-values for comparison between CKD patients and controls.

a *p* < 0.05 vs. CKD1-3 patients.

b *p* < 0.05 vs. CKD4-5 patients.

c *p* < 0.05 vs. HD patients.

BMI, body mass index; SBP, systolic blood pressure; DBP, diastolic blood pressure; ALK, alkaline phosphatase; PTH, parathyroid hormone; ESR, erythrocyte sedimentation rate; TSH, thyroid stimulating hormone; TC, total cholesterol; TG, triglycerides; LDH, lactose dehydrogenase.

### CMR scanning protocol

The CMR scans were performed in a 1.5 T MAGNETOM Aera MRI scanner (Siemens Healthcare, Erlangen, Germany) equipped with 18-channel phased-array surface coils using the vector electrocardiogram gating. Participants with CKD were all scanned on the next day after confirmation of CKD by nephrologists. Dialysis patients were all scanned on non-dialysis days but not after a long break; thus, all scans were performed within 18 to 24 h after the most recent dialysis session (16). Cine imaging of the LV long axis and sequential short-axis planes was performed using the balanced steady-state free precession (SSFP) sequence. Cine imaging parameters were as follows: repetition time, 2.93 ms; echo time, 1.16 ms; slice thickness, 6 mm; flip angle, 80°; field of view, 340 × 255 mm; matrix, 256 × 205; and 25 calculated cardiac phases.

Native T1 mapping at the base, mid, and apical levels of the LV short axis were performed using the modified look-locker inversion recovery (MOLLI) sequence. The T1 mapping parameters were as follows: repetition time, 3.89 ms; echo time, 1.12 ms; slice thickness, 8 mm; flip angle, 35°; field of view, 360 × 270 mm; and matrix, 256 × 192. T2 values of the LV myocardium were estimated from the T2 map that was generated using the T2-prepared single-shot SSFP sequence. T2 mapping parameters were as follows: repetition time, 3.244 ms; echo time, 1.35 ms; slice thickness, 8 mm; flip angle, 70°; field of view, 360 × 75 mm; and matrix, 192 × 83.

### Analysis of cardiac volume index and function

CMR image analysis was performed using the commercially available CVI42 software (Circle Cardiovascular, Calgary, Canada), and the analyst were blinded to the CKD group of study populations. The volumetric and functional parameters of the left ventricle were measured using the continuous short-axis slice cine images by manually tracing the endocardial and epicardial borders. The papillary muscles and trabeculations were excluded as part of the ventricular mass. CMR parameters such as LV end-diastolic volumes index (EDVI), end-systolic volume index (ESVI), stroke volume index (SVI), ejection fraction (EF), LV mass index (LVMI) and cardiac index (CI) were measured by the commercial CVI42 software (Circle Cardiovascular, Calgary, Canada) automatically. The left atrial volume index (LAVI) was calculated manually by tracing the endocardial left atrial (LA) borders in the two long-axis cine images (2 and 4-chamber views). The values were adjusted for the body surface area using the Mosteller formula (2) (BSA (m^2^) = √ ((weight (kg) × height (cm)/3600)).

### Estimation of the native T1 and T2 values

**Figure 1:**
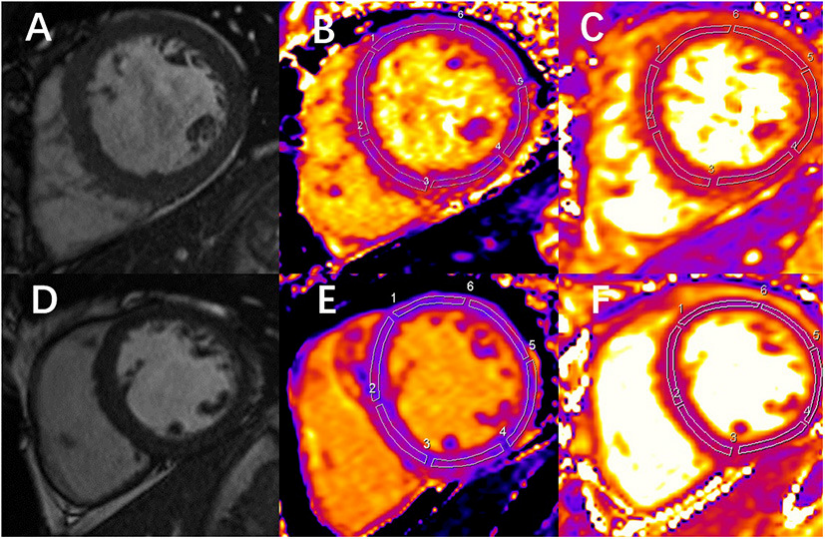
Examples of end-diastolic cine images and corresponding measurement of native T1 and T2 in 1 chronic kidney disease (CKD) patient and 1 healthy control. **(A)** Image shows the left ventricular middle short-axis segment. **(B,C)** Images are the measurement of native T1 and T2 mapping, respectively, at the same slice position in the same patient. **(A-C)** Images correspond to a 49-year-old CKD male patient. The mean global T1 value is 1,061 ms, and the mean global T2 value is 52 ms. **(D)** Image shows the left ventricular middle short-axis segment. **(E,F)** Images are the measurement of native T1 and T2 mapping, respectively, at the same slice position in the same person. **(D-F)** Images correspond to a 32-year-old healthy volunteer. The mean global T1 value is 1,003 ms, and the mean global T2 value is 45 ms.

The regions of interest (ROIs) were manually delineated in the mid-layer of the myocardium among the basal, middle, and apical LV segments to measure native T1 and T2 values. Sixteen ROIs were drawn for each participant based on the American Heart Association 16-segment model (Figures 1A–F). The image artifacts and coronary artery were eliminated from the ROIs. The average native T1 and T2 values were calculated from the three short-axis slices.

### Estimation of the myocardial systolic strain

**Figure 2:**
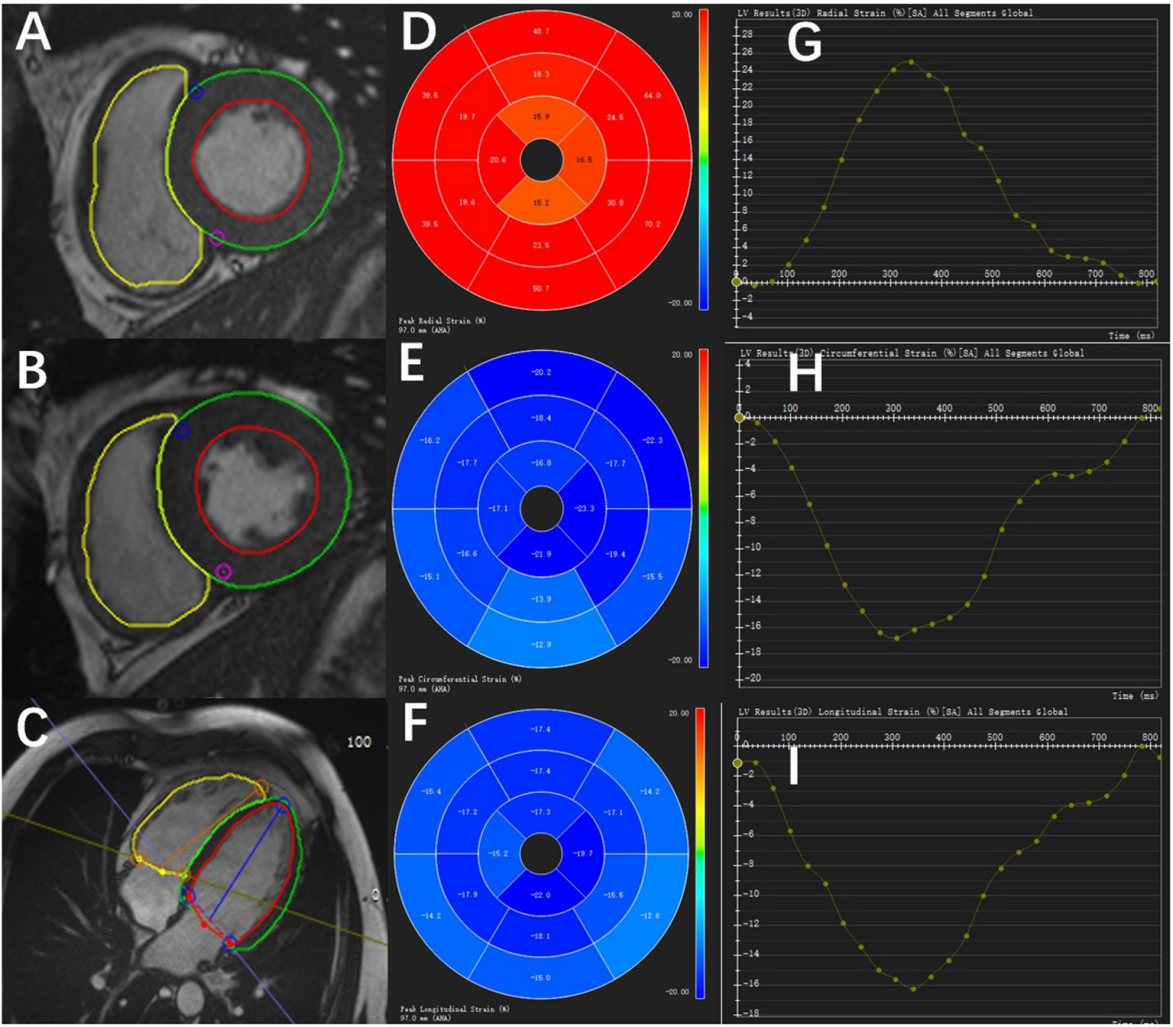
Diagram of the peak systolic strain analysis of the left ventricular myocardium in a healthy control by CVI42 software. The left shows the endocardial and epicardial borders of different short-axis slice cine images **(A,B)** and long-axis cine image **(C)**. In the middle is the 16-segment model of the radial **(D)**, circumferential **(E)**, and longitudinal **(F)** values in a cardiac cycle. On the right are the strain–time curves of the radial **(G)**, circumferential **(H)**, and longitudinal **(I)** values in a cardiac cycle.

The peak systolic LV strain parameters were calculated using the CVI42 software (Circle Cardiovascular, Calgary, Canada). Multiple long-axis cine images (2, 3, and 4-chamber views) and short-axis cine images were imported into the software. Then, the endocardial and epicardial borders of the LV were delineated in the end-diastolic frame (including papillary muscles and trabeculations) and automatically propagated throughout the cardiac cycle. The global longitudinal strain (GLS), global circumferential strain (GCS), and global radial strain (GRS) of the LV were obtained manually from the cine images (Figures 2A–I).

### Statistical analysis

Statistical analysis was performed using the SPSS version 26.0 software (SPSS Inc., Chicago, Illinois, USA). Kolmogorov–Smirnov test was used to analyze the normal distribution of continuous data. The normally and non-normally distributed data were summarized as means ± standard deviation and median (interquartile range, IQR), respectively. Differences between the normally distributed variables were analyzed using the independent-sample Student's *t*-test. Differences between the categorical variables were analyzed using the chi-square test. Differences in the clinical and CMR parameters between the three CKD patient groups and healthy subjects were compared by analysis of variance (ANOVA). Differences in the CMR variables after adjusting for age, BMI, and HR were assessed by analysis of covariance (ANCOVA). The relationships between CMR parameters and CKD stages of CKD patients without hemodialysis were examined using Spearman's correlation tests. *P*-value < 0.05 (two-tailed) was considered statistically significant.

## Results

### Basic clinical characteristics of the study groups

In this study, 84 participants with CKD and 32 healthy controls were enrolled. Table 1 shows the demographics of all the study subjects and the biochemical indices of the CKD patients. The basic characteristics including age (*p* = 0.336), sex (*p* = 0.220), BMI (*p* = 0.064) and HR (*p* = 0.205) were similar between the group of participants with CKD and healthy subjects. However, significant differences in age (*p* < 0.001), BMI (*p* = 0.023), and HR (*p* = 0.001) were observed among the healthy subjects and the three groups of participants with CKD, namely, CKD 1–3, CKD 4–5, and HD (Table 1). The healthy subjects did not show any history of cardiovascular diseases, hypertension, hyperlipidemia, and diabetes.

### Non-HD participants with CKD show significant alterations in left ventricular mass, volume, and function

Table 2 shows the LV mass, volume, and functional characteristics for the three CKD groups (I-III) and the healthy subjects. Table 3 shows the LV mass, volume, and functional parameters for the CKD patients without HD (groups I and II) and the healthy subjects after adjusting for age, BMI, and HR. The CMR-derived parameters LVEDVI, LVSVI, LVMI, and CI values were significantly increased starting from later stages (LVEDVI: 80 vs. 57 ml/m^2^; LVSVI: 42 vs. 32 ml/m^2^; LVMI: 57 vs. 36 g/m^2^; CI: 2.9 vs. 2.2 l/min/m^2^, *p* < 0.05 between group II and healthy subjects for all). Furthermore, the CMR-derived parameters LVESVI, LVEF and maximum LAVI values never had significant change in patients without HD.

**Table 2:** MRI characteristics of the study subjects.

**Variables**	**Healthy subjects (*n* = 32)**	**Participants with CKD (*****n*** = **84)**			***p*-values**
		**Group I (CKD 1-3, *n* = 23)**	**Group II (CKD 4-5, *n* = 20)**	**Group III (HD, *n* = 41)**	
LVEDV index (ml/m^2^)	57 ± 12	65 ± 16	81 ± 23	81 ± 29	**<0.001**
LVESV index (ml/m^2^)	26 ± 8	29 ± 10	39 ± 16	42 ± 23	**<0.001**
LVSV index (ml/m^2^)	32 ± 7	36 ± 10	42 ± 10	39 ± 12	**0.001**
LVEF (%)	55 ± 7	56 ± 10	54 ± 10	50 ± 10	**0.047**
LVM index (ml/m^2^)	36 ± 10	48 ± 19	58 ± 15	60 ± 21	**<0.001**
CI (l/min/m^2^)	2.1 ± 0.5	2.2 ± 0.7	3.0 ± 0.9	2.8 ± 0.9	**<0.001**
Max LAV index (ml/m^2^)	35 ± 12	34 ± 9	42 ± 12	45 ± 19	**0.002**
Min LAV index (ml/m^2^)	14 ± 8	13 ± 6	14 ± 4	21 ± 16	**0.006**
GRS (%)	37 ± 9	37 ± 15	30 ± 9	30 ± 11	**0.012**
GCS (%)	−21 ± 3	−20 ± 3	−19 ± 3	−18 ± 4	**0.001**
GLS (%)	−17 ± 3	−15 ± 3	−14 ± 3	−14 ± 4	**<0.001**
Global T1 (ms)	1006 ± 22	1024 ± 34	1045 ± 38	1055 ± 33	**<0.001**
Septal T1 (ms)	1005 ± 42	1030 ± 39	1054 ± 34	1065 ± 39	**<0.001**
Midseptal T1 (ms)	1014 ± 26	1034 ± 45	1058 ± 41	1068 ± 47	**<0.001**
Global T2 (ms)	47 ± 3	50 ± 3	53 ± 4	51 ± 3	**<0.001**
Septal T2 (ms)	46 ± 3	50 ± 3	53 ± 5	50 ± 3	**<0.001**
Midseptal T2 (ms)	46 ± 3	50 ± 4	54 ± 5	50 ± 4	**<0.001**

*p*-values for comparison among the four groups.

LVEDV, left ventricular end-diastolic volume; LVESV, left ventricular end-systolic volume; LVSV, left ventricular systolic volume; LVM, left ventricular mass; CI, cardiac index; Max LAV index, maximum left atrial volume index; Min LAV index, minimum left atrial volume index; GRS, global radial strain; GCS, global circumferential strain; GLA, global longitudinal strain. The bold values represent that *p* < 0.05 between groups.

**Table 3:** MRI characteristics of the study subjects after adjustments for age, BMI, and HR.

**Variables**	**Healthy subjects (*n* = 32)**	**CKD patients without HD (*****n*** = **43)**		**Start from which stage(s)**	***p*-values**
		**Group I (CKD 1–3, *n* = 23)**	**Group II (CKD 4–5, *n* = 20)**		
LVEDV index (ml/m^2^)	57 ± 4	60 ± 5	80 ± 5	Later	0.004
LVESV index (ml/m^2^)	26 ± 3	26 ± 4	38 ± 4	Never	/
LVSV index (ml/m^2^)	32 ± 2	35 ± 3	42 ± 3	Later	0.004
LVEF (%)	56 ± 2	58 ± 2	54 ± 2	Never	/
LVM index (g/m^2^)	36 ± 3	44 ± 4	57 ± 4	Later	<0.001
CI (l/min/m^2^)	2.2 ± 0.1	2.4 ± 0.2	2.9 ± 0.2	Later	0.006
Max LAV index (ml/m^2^)	35 ± 3	33 ± 4	43 ± 4	Never	/
GRS (%)	37 ± 2	38 ± 3	30 ± 3	Never	/
GCS (%)	−20.6 ± 0.5	−20.2 ± 0.7	−18.9 ± 0.7	Never	/
GLS (%)	−16.2 ± 0.5	−15.1 ± 0.6	−13.7 ± 0.7	Later	0.024
Global T1 (ms)	1009 ± 6	1027 ± 7	1041 ± 7	Later	0.003
Septal T1 (ms)	1006 ± 7	1033 ± 9	1052 ± 9	Later	0.001
Midseptal T1 (ms)	1017 ± 8	1037 ± 9	1053 ± 10	Later	0.017
Global T2 (ms)	46.6 ± 0.5	49.9 ± 0.6	53.2 ± 0.7	Early	<0.001
Septal T2 (ms)	46.1 ± 0.6	49.5 ± 0.7	52.6 ± 0.7	Early	0.002
Midseptal T2 (ms)	46.1 ± 0.6	50.3 ± 0.8	53.3 ± 0.8	Early	<0.001

The column named “start from which stage(s)” represents the stage(s) of CKD in patients who developed significant abnormalities compared to healthy subjects: “early” meaning *p* < 0.05 between control and group I; “later” meaning p only <0.05 between control and group II, in the other words, *p* ≥ 0.05 between control and group I; and “never” meaning *p* ≥ 0.05 among all groups.

*p*-values for comparison between patients who developed significant abnormalities and healthy subjects.

BMI, body mass index; HR, heart rate; LVEDV, left ventricular end-diastolic volume; LVESV, left ventricular end-systolic volume; LVSV, left ventricular systolic volume; LVM, left ventricular mass; CI, cardiac index; Max LAV index, maximum left atrial volume index; Min LAV index, minimum left atrial volume index; GRS, global radial strain; GCS, global circumferential strain; GLA, global longitudinal strain.

### Non-HD participants with CKD show significant alterations in the left ventricular strain

**Figure 3:**
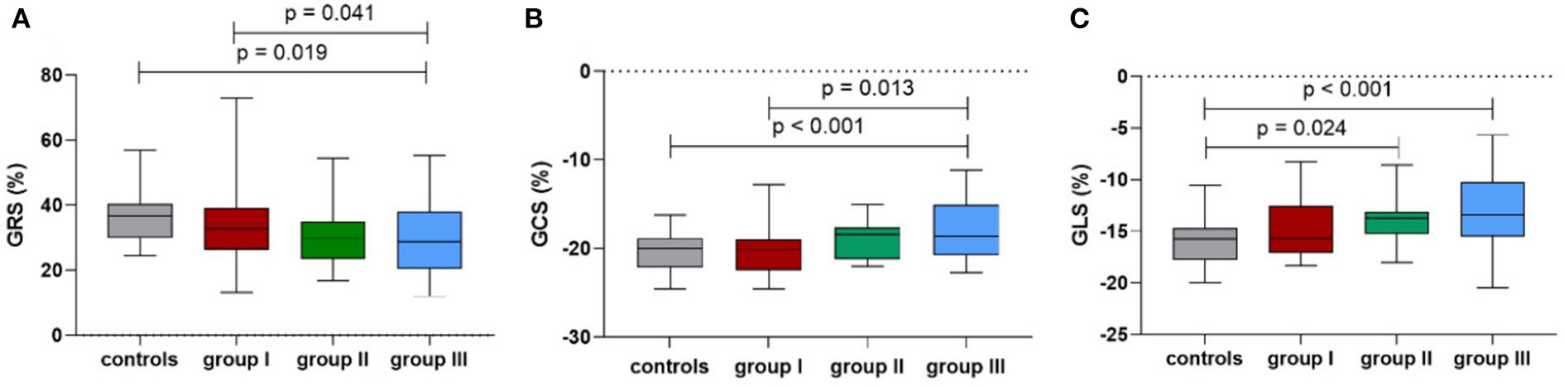
Comparison of the mean peak global radial strain **(A)**, peak global circumferential strain **(B)** and peak global longitudinal strain **(C)** among the controls and groups I-III.

Table 3 summarizes the values for the LV strain parameters in the three groups after adjusting for age, BMI, and HR. The CMR-derived parameters GRS and GCS never had significant change in patients without HD (Figures 3A,B). However, the CMR-derived parameter GLS values were significantly reduced starting from later stages (group II vs. healthy subjects: −13.7 ± 0.7 vs. −16.2 ± 0.5, *p* = 0.024) (Figure 3C).

### Non-HD participants with CKD show significant alterations in myocardial native T1 and T2 values

**Figure 4:**
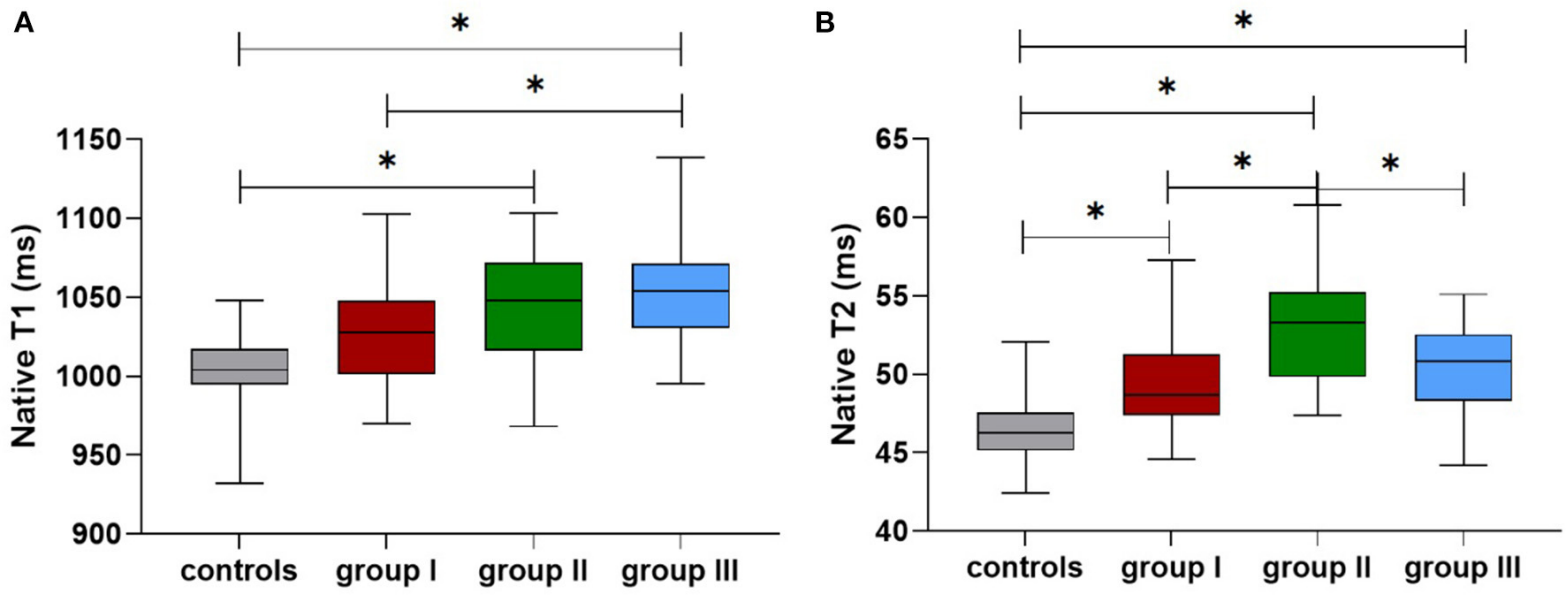
Comparison of the native T1 **(A)** and T2 values **(B)** among the controls and groups I-III (*means that *p* < 0.05 between the two groups).

Table 3 shows the differences in the native T1 and T2 values between the three groups after adjusting for age, BMI, and HR. The global cardiac native T1 values were higher in the CKD groups compared with the healthy subjects; the values showed an incremental increase with CKD severity (healthy subjects: 1,009 ± 6; CKD group I: 1,027 ± 7; CKD group II: 1,041 ± 7). The CMR-derived parameter global native T1 values were significantly increased starting from later stages (group II vs. control: *p* = 0.003) (Figure 4A). Furthermore, the global T2 values were significantly increased starting from early stages (group I vs. healthy subjects: 49.9 ± 0.6 vs. 46.6 ± 0.5, *p* < 0.001) (Figure 4B). The septal and midseptal T1 and T2 values and the inter-group differences showed similar trends as observed with the global T1 and T2 values, respectively (Table 3).

### Left ventricular CMRI parameters and native T1 and T2 values correlate with advanced CKD stage (stages 1–5)

**Figure 5:**
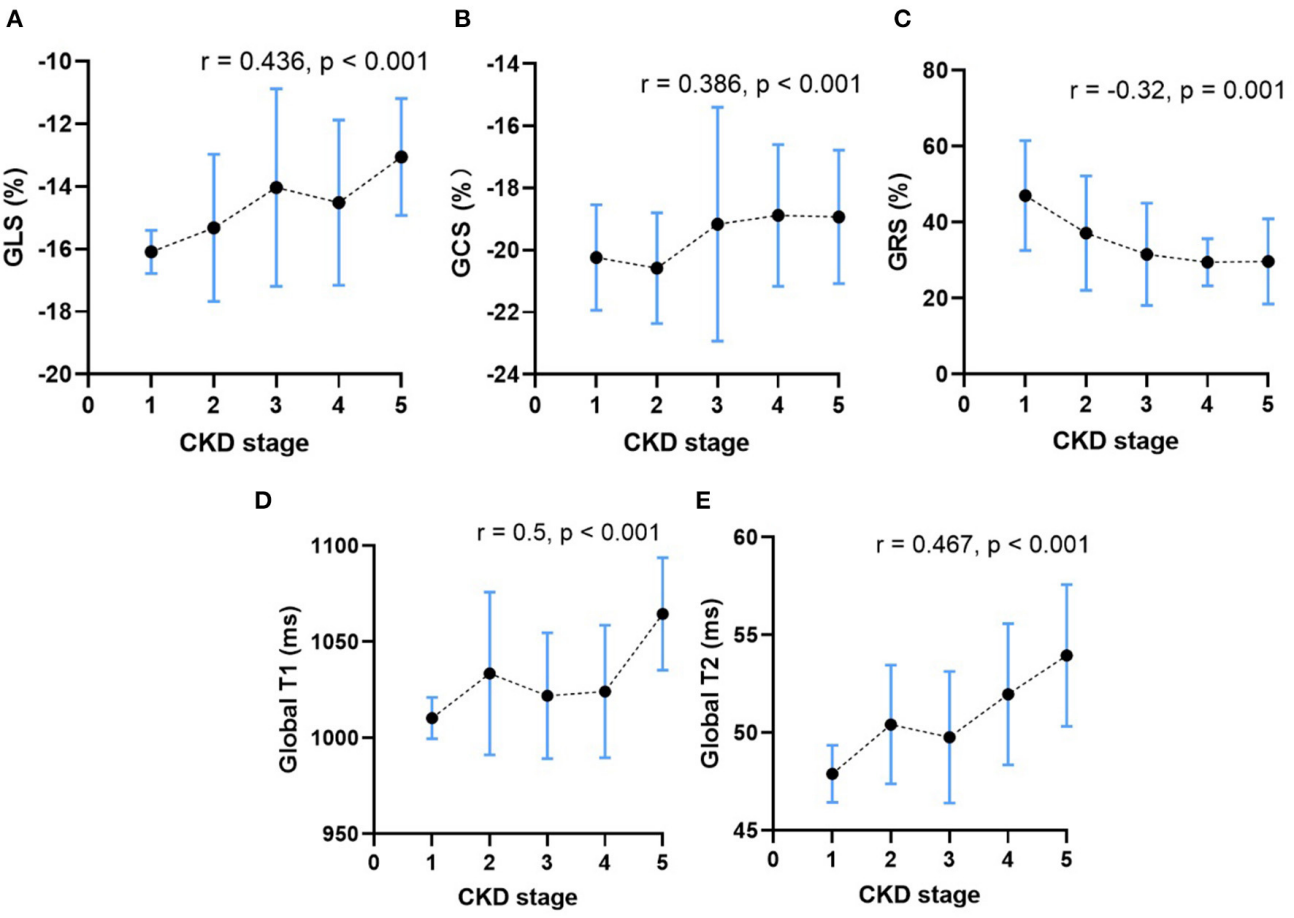
The relationship between CKD stage and the mean peak global radial strain **(A)**, peak global circumferential strain **(B)**, peak global longitudinal strain **(C)**, the global native T1 values **(D)** and the global T2 values **(E)** in CKD patients without HD. The dots represent the mean CMR parameters of patients in each CKD stage, and the vertical bars represent the standard deviation range of CMR parameters of patients in each CKD stage. The dashed line represents the trends of change in CMR parameters.

As shown in Table 4, CKD stages showed inverse correlation with BMI (*r* = −0.246, *p* = 0.008) and positive correlation with HR (*r* = 0.29, *p* = 0.002). After adjusting for BMI and HR, CKD stages of patients without HD showed positive correlation with the LVEDVI (*r* = 0.425, *p* < 0.001), LVESVI (*r* = 0.405, *p* < 0.001), LVMI (*r* = 0.535, *p* < 0.001), CI (*r* = 0.293, *p* = 0.002), GLS (*r* = 0.436, *p* < 0.001), and GCS (*r* = 0.386, *p* < 0.001) (Figures 5A,B). Furthermore, CKD stages of patients without HD showed weak negative correlation with LVEF (*r* = −0.255, *p* = 0.006) and GRS (*r* = −0.32, *p* = 0.001) (Figure 5C). Furthermore, CKD stages of patients without HD showed significant positive correlation with the global native T1 (*r* = 0.5, *p* < 0.001) and T2 values (*r* = 0.467, *p* < 0.001) (Figures 5D,E).

**Table 4:** Univariate correlation coefficients for worsening CKD stage.

**Variables**	**CKD stage**		**Adjusted for BMI and HR**	
	***R*-value**	***P*-value**	***R*-value**	***P*-value**
Age (years)	0.101	0.281	0.106	0.263
Male (*n*, %)	0.126	0.178	0.198	0.035
BMI (kg/m2)	−0.246	**0.008**	-	-
HR (bpm)	0.29	**0.002**	-	-
LVEDV index (ml/m2)	0.426	**<0.001**	0.425	**<0.001**
LVESV index (ml/m2)	0.402	**<0.001**	0.405	**<0.001**
LVSV index (ml/m2)	0.309	**0.001**	0.299	**0.001**
LVEF (%)	−0.238	**0.01**	−0.255	**0.006**
LVM index (ml/m2)	0.52	**<0.001**	0.535	**<0.001**
CI (l/min/m2)	0.411	**<0.001**	0.293	**0.002**
Max LAV index (ml/m2)	0.317	**0.001**	0.304	**0.001**
GRS (%)	−0.308	**0.001**	−0.32	**0.001**
GLS (%)	0.424	**<0.001**	0.436	**<0.001**
GCS (%)	0.372	**<0.001**	0.386	**<0.001**
Global T1 (ms)	0.567	**<0.001**	0.5	**<0.001**
Septal T1 (ms)	0.556	**<0.001**	0.502	**<0.001**
Midseptal T1 (ms)	0.498	**<0.001**	0.431	**<0.001**
Global T2 (ms)	0.485	**<0.001**	0.467	**<0.001**
Septal T2 (ms)	0.426	**<0.001**	0.431	**<0.001**
Midseptal T2 (ms)	0.407	**0.007**	0.386	**<0.001**

CKD, chronic kidney disease; BMI, body mass index; HR, heart rate; LVEDV, left ventricular end-diastolic volume; LVESV, left ventricular end-systolic volume; LVSV, left ventricular systolic volume; LVM, left ventricular mass; CI, confidence interval; Max LAV index, maximum left atrial volume index; GRS, global radial strain; GCS, global circumferential strain; GLA, global longitudinal strain. The bold values represent that *p* < 0.05 between groups.

### Comparison of CMR parameters between HD patients and each other groups

Table 5 shows comparison of CMR parameters between HD patients and each other groups (healthy subjects, group I and group II). All CMR parameters in HD patients were significantly worse than those in healthy subjects. In addition, LVEF and LV strain parameters of HD patients were worse than those of CKD patients in group II (stages 4–5 without HD) (LVEF: 49 vs. 54%; GRS: 29 ± 2 vs. 30 ± 3; GCS: −17.4 ± 0.5 vs. −18.9 ± 0.7; GLS: −12.9 ± 0.5 vs. −13.7 ± 0.7), however, the above parameters comparison were not significant. Furthermore, the global native T1 values were higher in the HD patients compared with group II patients (1,053 ± 6 vs. 1,041 ± 7, *p* > 0.05). In contrast, T2 values in the HD patients were significantly lower compared with those non-HD patients with CKD stages 4-5 (50.1 ± 0.5 vs. 53.2 ± 0.7, *p* = 0.002) (Figures 4A,B). The septal and midseptal T1 and T2 values and the inter-group differences for these values were similar to the trends observed for the global T1 and T2 values, respectively.

**Table 5:** Comparison of CMR characteristics between HD patients and each other groups after adjustments for age, BMI, and HR.

**Variables**	**Healthy subjects** **(*n* = 32)**	**Group I (CKD 1-3, *n* = 23)**	**Group II (CKD 4-5, *n* = 20)**	**Group III (HD, *n* = 41)**	**^#^*p*-values**	**^$^*p*-values**	**^&^*p*-values**
LVEDV index (ml/m^2^)	57 ± 4	60 ± 5	80 ± 5	84 ± 4	**<0.001**	**0.002**	1.000
LVESV index (ml/m^2^)	26 ± 3	26 ± 4	38 ± 4	45 ± 3	**<0.001**	**0.001**	0.941
LVSV index (ml/m^2^)	32 ± 2	35 ± 3	42 ± 3	40 ± 2	**0.009**	0.719	1.000
LVEF (%)	56 ± 2	58 ± 2	54 ± 2	49 ± 2	**0.021**	**0.006**	0.248
LVM index (g/m^2^)	36 ± 3	44 ± 4	57 ± 4	63 ± 3	**<0.001**	**0.002**	1.000
CI (l/min/m^2^)	2.2 ± 0.1	2.4 ± 0.2	2.9 ± 0.2	2.7 ± 0.1	**0.013**	0.520	1.000
Max LAV index (ml/m^2^)	35 ± 3	33 ± 4	43 ± 4	45 ± 3	**0.016**	**0.023**	1.000
GRS (%)	37 ± 2	38 ± 3	30 ± 3	29 ± 2	**0.019**	**0.041**	1.000
GCS (%)	−20.6 ± 0.5	−20.2 ± 0.7	−18.9 ± 0.7	−17.4 ± 0.5	**<0.001**	**0.013**	0.564
GLS (%)	−16.2 ± 0.5	−15.1 ± 0.6	−13.7 ± 0.7	−12.9 ± 0.5	**<0.001**	0.069	1.000
Global T1 (ms)	1009 ± 6	1027 ± 7	1041 ± 7	1053 ± 6	**<0.001**	**0.036**	1.000
Septal T1 (ms)	1006 ± 7	1033 ± 9	1052 ± 9	1063 ± 7	**<0.001**	0.065	1.000
Midseptal T1 (ms)	1017 ± 8	1037 ± 9	1053 ± 10	1066 ± 7	**<0.001**	0.113	1.000
Global T2 (ms)	46.6 ± 0.5	49.9 ± 0.6	53.2 ± 0.7	50.1 ± 0.5	**<0.001**	1.000	**0.002**
Septal T2 (ms)	46.1 ± 0.6	49.5 ± 0.7	52.6 ± 0.7	49.6 ± 0.5	**<0.001**	1.000	**0.007**
Midseptal T2 (ms)	46.1 ± 0.6	50.3 ± 0.8	53.3 ± 0.8	49.6 ± 0.6	**0.001**	1.000	**0.001**

# *p*-values for comparison between healthy subjects and HD patients.

$ *p*-values for comparison between patients with CKD 1–3 and HD patients.

& *p*-values for comparison between patients with CKD 4–5 and HD patients. The bold values represent that *p* < 0.05 between groups.

## Discussion

This study shows that CMR parameters reflecting LV myocardial structure and function get worsen with advancing CKD stages. Participants with CKD stages 4–5 were more likely to occur significantly increased T1 values, which may reflect myocardial fibrosis. The increased native T1 values and decreased T2 values of HD patients might be due to increasing myocardial fibrosis in the HD group but with reduction in oedema following effective fluid management on dialysis.

In this prospective study, changes in the myocardial strain parameters like GLS, GCS, and GRS were associated with advanced CKD stage. This suggested significant alterations in the myocardial wall remodeling among patients with advanced or severe CKD. Furthermore, this study showed that GLS of participants with CKD decreased significantly from stages 4–5 onwards when compared with the healthy subjects, whereas LVEF values were reduced in the CKD patients undergoing hemodialysis. These results were in agreement with previous reports demonstrating GLS as a more sensitive parameter than LVEF in detecting subclinical LV dysfunction because GLS directly measures the movement of myocardial fibers (9, 17). The prognostic value of GLS was also higher than the values for LVEF in predicting mortality and adverse cardiovascular outcomes (18, 19). Therefore, CKD patients with reduced GLS require close monitoring for early detection of cardiac abnormalities.

A previous report (9) suggested that aberrant GLS was secondary to myocyte hypertrophy caused by myocardial fibrosis. Our study shows higher native T1 and T2 values in CKD patients compared with healthy subjects, and increasing native T1 and T2 values with the advancing CKD stages, which are consistent with previous studies (8, 18). In addition, a negative correlation between T2 values and GFR was also found in patients with heart failure (20), supporting the finding of our trial. Other previous studies demonstrated that native T1 mapping improved the diagnostic accuracy of CMR in estimating myocardial fibrosis (16, 21). In addition, myocardial native T1 values were also associated with myocardial edema, infiltration of immune cells (22), acute myocardial infarction or amyloidosis (23). Participants enrolled in our study had no history of heart disease or amyloidosis, suggesting that the elevated T1 values in participants with advanced CKD were irrelevant to based heart disease or amyloidosis. However, we cannot rule out the effect of myocardial edema on the changes of T1 values. Native T2 mapping measures the free water content in tissues including the cardiac tissue (7). In current study, myocardial native T1 values and T2 values of CKD groups are higher than healthy subjects showing that myocardium of CKD patients may develop both fibrosis and oedema. Furthermore, our results demonstrate that CKD patients may develop worsening myocardial fibrosis and oedema with advancing CKD severity.

CKD progression facilitates the onset of myocardial fibrosis (24). However, the exact CKD stage at which myocardial fibrosis is clinically manifest is not well-characterized. In the current study, participants with CKD stages 4–5 showed significantly higher native T1 and T2 values compared with healthy subjects. These results supported previous biopsy findings from ESRD patients that showed extensive interstitial fibrosis (25, 26). Edwards et al. (11) reported irreversible fibrosis in 14% of stage 2 to 4 CKD patients using late gadolinium enhancement (LGE). These slightly different results may be explainable by differences in sample size and their use of gadolinium. LGE is not sensitive enough to detect diffuse myocardial fibrosis (24) and has risk of nephrogenic systemic fibrosis (27). Our results suggested that participants with CKD stages 4–5 were more likely to develop significant myocardial fibrosis. However, further investigations and more sensitive magnetic resonance imaging (MRI) techniques are needed to determine if myocardial fibrosis occurs in patients with early CKD.

In our study, participants with CKD undergoing HD showed significantly reduced T2 values compared with non-HD patients with CKD stages 4–5. Previous studies reported that T2 values were related to myocardial fluid content (28–30). Our results showed that myocardial oedema may be improved in ESRD patients undergoing HD compared with non-HD patients with advanced CKD, thereby supporting previous findings (7, 31). Kotecha et al. (7) showed that native T1 and T2 values were significantly reduced in ESRD patients after dialysis, suggesting that these values reflected an acute reduction of myocardial water content. Rankin et al. (31) also showed significant decrease in native T1 and T2 values following hemodialysis with fluid removal in CKD patients undergoing HD based on 3T CMR. However, the changes of T1 values in our current study may seem contradictory to these two studies. The reason for this difference may be that Kotecha and Rankin et al. referred to within-subject differences pre/post HD. T1 values of participants with CKD undergoing HD in this study increased slightly than non-HD patients with CKD stages 4–5. This suggested that for CKD patients undergoing HD, myocardial fibrosis, rather than myocardial oedema, accounts for the elevated T1 values. Similarly, Graham-Brown et. compared 124 HD patients to 137 healthy subjects and found that the obviously elevated T1 values occurred independently with T2 values, which had no significant changes. It is reasonable to speculate that the observed increase in native T1 values and decrease in T2 values between CKD 4–5 and HD patients might be due to increasing fibrosis in the dialysis group but with reduction in oedema following effective fluid management on dialysis. However, our study did not compare native T1 values of CKD patients before and after dialysis. Hence, this aspect requires further investigation.

Our study has a few limitations. Firstly, the sample size of our single-center study was small. It was difficult to stratify patients into CKD 1-5 stage and directly state which stage of 1-5 shows the significant reduction in myocardial function. Therefore, large cohort, multi-center studies are necessary to confirm our findings and explore potential relationships between myocardial abnormalities and CKD stages. Secondly, the cross-sectional nature of this study may have affected the results because of individual differences among the study subjects. We did not compare parameters of participants with CKD before and after dialysis. Therefore, in the future, a longitudinal study needs to be performed to confirm our results. Thirdly, although the CKD groups were matched with the healthy subjects based on age, BMI, and HR, significant inter-group differences existed among the four groups. Therefore, we used ANCOVA to exclude the influence of these confounding factors. Forthly, there was a lack of assessment of myocardial fibrosis and fluid status, either clinically or bioimpedance. Finally, because of the small sample size, we did not investigate the effects of important factors such as hypertension and diabetes that can influence cardiac functions.

## Conclusions

In conclusion, myocardial strain, native T1, and T2 values progressively got worse as the severity of CKD increased. Myocardial fibrosis and edema were observed more frequently in participants with advanced CKD or severe CKD requiring HD. The increased native T1 values and decreased T2 values of CKD patients undergoing HD might be due to increasing myocardial fibrosis in the HD group but with reduction in oedema following effective fluid management on dialysis. Further large cohort multi-center studies are needed to confirm our findings.

## Data availability statement

The raw data supporting the conclusions of this article will be made available by the authors, without undue reservation.

## Ethics statement

The studies involving human participants were reviewed and approved by the Ethics Committee of the Tongji Medical College of Huazhong University of Science and Technology. The patients/participants provided their written informed consent to participate in this study.

## Author contributions

XJ, XH, and YC conceived and designed the study. YC and HS provided the administrative support. YW, FH, and CZ provided study materials and patients. XJ, XH, and YZ collected and assembled data of the study. XJ and XH analyzed and interpreted the study data. All authors read and approved the final version of the manuscript.

## Conflict of interest

Author XZ was employed by MR Collaborations, Siemens Healthineers Digital Technology (Shanghai) Co., Ltd., Shanghai, China. The remaining authors declare that the research was conducted in the absence of any commercial or financial relationships that could be construed as a potential conflict of interest.

## Publisher's note

All claims expressed in this article are solely those of the authors and do not necessarily represent those of their affiliated organizations, or those of the publisher, the editors and the reviewers. Any product that may be evaluated in this article, or claim that may be made by its manufacturer, is not guaranteed or endorsed by the publisher.

## Abbreviations

CKD: chronic kidney disease
CMR: cardiac magnetic resonance
ANCOVA: analysis of covariance
LVEF: left ventricular ejection fraction
GRS: global radial strain
GCS: global circumferential strain
GLS: global longitudinal strain
CVD: cardiovascular disease
eGFRs: estimated glomerular filtration rates
HD: hemodialysis
LV: left ventricular
ESRD: end-stage renal disease
BMI: body mass index
MDRD: Modification of Diet in Renal Disease
HR: heart rate
SBP: systolic blood pressure
DBP: diastolic blood pressure
AKP: alkaline phosphatase
PTH: parathyroid hormone
ESR: erythrocyte sedimentation rate
TSH: thyroid-stimulating hormone
FT3: free triiodothyronine
FT4: free tetraiodothyronine
TG: triglycerides
TC: total cholesterol
LDH: lactate dehydrogenase
SSFP: steady-state free precession
MOLLI: modified look-locker inversion recovery
EDVI: end-diastolic volumes index
ESVI: end-systolic volume index
SVI: stroke volume index
EF: ejection fraction
LVMI: LV mass index
CI: cardiac index
LAVI: The left atrial volume index
LA: left atrial
BSA: body surface area
ROIs: the regions of interest
IQR: interquartile range
ANOVA: analysis of variance
LGE: late gadolinium enhancement
MRI: magnetic resonance imaging.
